# Color coded perfusion imaging with contrast enhanced ultrasound (CEUS) for post-interventional success control following trans-arterial chemoembolization (TACE) of hepatocellular carcinoma

**DOI:** 10.1371/journal.pone.0217599

**Published:** 2019-06-10

**Authors:** Janine Rennert, Isabel Wiesinger, Andreas Schicho, Lukas Philip Beyer, Philipp Wiggermann, Christian Stroszczynski, Ernst Michael Jung

**Affiliations:** University Hospital Regensburg, Department of Radiology, Regensburg, Germany; Texas A&M University, UNITED STATES

## Abstract

**Aim:**

Evaluation of an external color coded perfusion quantification software with CEUS for the post-interventional success control following TACE in patients with HCC.

**Material and methods:**

31 patients (5 females, 26 males, age range 34–82 years, mean 66.8 years) with 59 HCC lesions underwent superselective TACE using DSM Beads between 01/2015 and 06/2018. All patients underwent CEUS by an experienced examiner using a convex multifrequency probe (1–6 MHz) within 24 hours following TACE to detect residual tumor tissue. Retrospective evaluation using a perfusion quantification software regarding pE, TTP, mTT, Ri and WiAUC in the center of the lesion, the margin and surrounding liver.

**Results:**

In all lesions, a post-interventional visual reduction of the tumor microvascularization was observed. Significant differences between center of the lesion vs. margin and surrounding liver were found regarding peak enhancement (867.8 ± 2416 center vs 2028 ± 3954 margin p<0.005) and center 867.8 ± 2416 vs 2824 ± 4290 surrounding liver, p<0.0001)). However, no significant differences were found concerning Ri, WiAuC, mTT and TTP.

**Conclusion:**

CEUS with color- coded perfusion imaging is a valuable supporting tool for post-interventional success control following TACE of liver lesions. Peak enhancement seems to be the most valuable parameter.

## Introduction

Liver lesions are the sixth most common cancer (approximately 750,000 new cases per year) and currently represent the third most common cause of cancer-related deaths worldwide. Hepatocellular carcinoma (HCC) shows an increasing incidence and generates over 90% of all primary hepatic cancers, thus representing a major healthcare problem [1].

HCC has a strong male predominance with an estimated male to female ratio of 2.4 [1]. Approximately 90% of HCCs are associated with known underlying risk factors. The most frequent factors include chronic viral hepatitis (types B and C), alcohol intake and aflatoxin exposure [2].

Hepatic cirrhosis is an important risk factor for HCC, commonly caused by chronic viral hepatitis, alcohol, inherited metabolic diseases such as hemochromatosis or alpha-1-antitrypsin deficiency, and non-alcoholic fatty liver disease. All etiologic forms of cirrhosis may be complicated by tumor formation, but the risk is higher in patients with hepatitis with 2–8% per year [3]. Various published guidelines agree on a typical vascular pattern of HCC lesions, being characterized by early arterial enhancement followed by contrast media wash-out beginning in the venous phase [4–6]. Small tumor lesions < 1 cm often display no venous wash out at all. The imaging modalities recommended for diagnosis of HCC include contrast enhanced magnetic resonance imaging (MRI), multi-phase contrast enhanced computed tomography (CT) and contrast enhanced ultrasound (CEUS). Current studies have shown a sensitivity for detection of HCC of up to 91.1% for CEUS [7–9] up to 87.5% for CT [10] and up to 95.9% for Gd-EOB-DTPA-Enhanced Liver MRI (Gadolinium ethoxybenzyl diethylenetriamine pentaacetic acid) [11].

Depending on the local tumor extent, extrahepatic spread and the established degree of liver damage, various therapeutic options are available for each stage. For early stage HCC surgical resection [12], ablation [13] and transplantation [14] are recommended. Furthermore trans-arterial chemoembolization (TACE) [15] is suggested for intermediate-stage HCC, sorafenib for advanced-stage HCC [16]. Finally, for terminal-stage HCC best supportive care is advised [5].

TACE is commonly used in two settings, either in HCC unsuitable for resection or ablation or liver transplantation as a bridge therapy [17]. The aim of TACE is to pad the tumor with a chemotherapeutic drug (eg, doxorubicin, epirubicin, cisplatin, or mitomycin C) using a carrier agent. Formerly, Lipiodol was used as the carrier agent, but this has been largely replaced by drug-eluting beads, which are available in different sizes [18].

Aim of this study was the evaluation of an external color coded perfusion quantification software with CEUS for the post-interventional success control following TACE in patients with HCC.

## Material and methods

### Study design

From January 2015 until June 2018, 31 patients (26 males, 5 females, age 34–82 years, mean 62.8 years) with 59 HCCs, identified by characteristic imaging features or histopathology were included in this retrospective study. Each patient underwent pre-interventional contrast-enhanced computed tomography (CT) and liver specific contrast enhanced magnetic resonance imaging (MRI) for detection and characterization of the liver lesions within 72 hours prior to the intervention. The study was approved by the University medical Center Regensburg ethics Committee (reference number 15-104-0115).

The therapeutic interventional procedure for each patient was a superselective trans-arterial chemoembolization (TACE) using degradable starch microspheres (DSM; EmboCept S, PharmaCept, Germany) loaded with 50 mg Epirubicin (Famorubicin, Pfizer, USA). The indication for a TACE was formed by an interdisciplinary tumor conference.

Each patient was examined using B- mode, Color Coded Doppler Sonography (CCDS), Power Doppler and Contrast Enhanced Ultrasound (CEUS). Before the imaging procedures were conducted, written informed consent was obtained from each patient for MRI, CT and CEUS. Exclusion criteria of this study were contraindications for use of a contrast agent for CT, MRI or CEUS, impaired renal function (creatinine >1.5 mg/dl, creatinine clearance < 30 ml/min), pre-existing strong allergic reactions and decompensated cardiac failure.

### Imaging studies

#### Basic ultrasound examination

Within 24 hours following TACE, ultrasound was performed by an experienced radiologist with national DEGUM stage III using a multifrequency convex transducer (1–6 MHz, LOGIQ E9, GE Healthcare).

First, the whole liver was examined using B-mode sonography in sweep technique. Color Coded Doppler Sonography (CCDS) and Power Doppler (PD) ultrasound were used to evaluate macro vascularization. Flow parameters were adjusted to the lowest possible pulse repetition frequency (PRF < 1000 Hz) and the best possible color imaging without blooming artifacts.

#### CEUS

Contrast enhanced ultrasound (CEUS) was performed after bolus injections of 1–2.4 ml of sulphur hexaflouride microbubbles (SonoVue, BRACCO, Italy) with a low mechanical index (MI < 0.16) applying CEUS with amplitude modulation and pulse inversion harmonic imaging (PIHI) technique. The contrast harmonic imaging technique (CHI) uses a contrast-specific detection mode for real-time evaluation of the contrast-agent enhancement. The complete data of the contrast-agent examination was recorded up to 5 min. The liver microcirculation was evaluated continuously from an early arterial phase (beginning 15 sec. after contrast application) until a late parenchymal phase (> 5 min.). The transducer was held steadily on the post-embolisation defect of 1 minute to avoid repeated injection of contrast media, afterwards the whole liver was examined in sweep technique to look for wash-out. The first minute was documented a video clip. Afterwards single images were stored until the late phase. All images were digitally stored in PACS.

Irregular enhancement in the periphery of the embolized area during early arterial phase, ideally combined with a wash-out starting during portal venous phase was seen as a characteristic criterion for residual HCC tissue. A uniform peripheral rim enhancement without wash-out was considered as physiological postembolization reaction. Wedge-shaped, homogenous arterial enhancement peripheral to the embolized area with progressively enhancing portal-venous branching but without washout was defined as arterio-portal-venous shunt.

CEUS was performed within 24 hours prior and following the procedure.

#### CT/ MRI

Each patient received a pre- and post-interventional contrast-enhanced dual source CT (ceCT) of the liver in arterial phase (25–35 sec), in portal-venous phase (70–90 sec) and late phase (>120 sce) with bolus injection of iodic contrast-agent (100–130 ml Accupaque, GE) (SOMATOM Definition Flash Siemens Healthcare, Erlangen Germany; collimation 5 mm with coronary and axial reconstructions). Before the treatment every patient received a ceCT of the whole abdomen/ pelvis in portal-venous phase to exclude extrahepatic tumor manifestation. Within 24 hours after the treatment only the liver was scanned in arterial and portal-venous phase to exclude post-interventional complications.

An additional pre-interventional contrast-enhanced MRI (ceMRI) was performed (Skyra 3T, Siemens Healthcare, Erlangen, Germany), using T1/ T2 sequences, diffusion imaging with ADC as well as contrast-enhanced sequences after bolus injection of 15–35 ml liver specific contrast agent (Gd-EOB-DTPA), (Primovist, Bayer, Schering Pharma AG, Germany) using 3D vibe sequences from arterial phase (20–25 sec) up to late phase.

For follow-up, patients received CEUS, MRI and CT scans according to the protocols mentioned above for up to 6 months.

#### Perfusion analysis

The digitally stored ultrasound loops were evaluated using a color coded perfusion quantification software (VueBox, Bracco, Italy) on a separate computer. DICOM loops were uploaded and opened in the VueBox platform (BRACCO, Italy) for blinded and independent reading. VueBox is a color-coded off-line general-purpose perfusion software for dynamic CEUS examinations, that uses automatic in-plane motion correction [19]. Four parameters were calculated for each Region of interest (ROI) which included time to peak (TTP), mean transit time (mTT), peak enhancement (pE), Wash-in Area Under the Curve (WiAUC) and Rise (Ri). Flow parameters such as regional blood volume and regional blood flow were calculated by the software and exported in a calculation protocol.

Regions of Interst (ROI) were manually placed in the center of the lesion, at the margins and the surrounding liver tissue > 2cm distance. The VueBox screen is divided into four quadrants: in the upper left quadrant the original image with the ROIs is displayed, in the upper right quadrant the corresponding parametric image is displayed, in the lower left quadrant the corresponding time/intensity curves are shown in the color corresponding to the ROI in the image above and in the lower right quadrant the respective numerical values of the selected curve parameter is shown [20, 21] (image 2).

### Imaging analysis

CEUS examinations and the perfusion analysis were read by two experienced radiologists in consensus. For each modality, each observer recorded the diagnostic findings. Furthermore, the image quality was documented on a four points scale: 1—excellent, 2—minor diagnostic limitations, 3 –major diagnostic limitations, 4—non-diagnostic.

Imaging modalities were evaluated using the data analysis hard-/software of the ultrasound system (LOGIQ E9, GE).

### Statistical analysis

For data analysis, IBM-SPSS software (version 19.0, SPSS Inc., Chicago, USA) was used.

For calculation of the differences between the center of the lesion and the margins, the margins and the surrounding liver as well as the center of the lesion and the surrounding liver for each parameter repeated measures ANOVA with Bonferroni post test was performed using GraphPad Prism version 5.00 for Mac OS X, GraphPad Software, San Diego California USA with alpha = 0.05 indicating statistically significant differences between groups.

## Results

### Lesions characteristics and tumor size

Each of the 59 tumor lesions showed an enhancement pattern consistent with hepatocellular carcinoma (HCC) including arterial irregular hyperenhancement and portal venous washout on CEUS. Also, the pre-interventional imaging techniques showed characteristics compatible with HCC. All 31 patients underwent pre-interventional MRI using a liver specific contrast agent (Primovist, Bayer HealthCare AG, Germany; 0.1 ml/kg body weight) and a pre-interventional CT. Also, post-interventional CT scans were performed within 24 hours after the procedure to identify complications. During the 6 months follow-up, all patients with recurrent disease were correctly identified using CEUS.

The pre-interventional tumor sizes of the 59 lesions ranged from 9 mm up to 55 mm with a mean size of 36 mm. The post-interventional defects sized from 11 mm to 55 mm with am mean size of 31 mm. In all lesions, a post-interventional reduction of the tumor microvascularization was observed.

### Perfusion analysis and CEUS

In all 31 patients (100%) CEUS was viable. The image quality in all examinations was excellent or had only minor diagnostic limitations (1–2 SD ± 0.372).

The data acquired for pE, WiAuC, mTT, TTP and Ri is shown in Table 1. No significant differences between center of the lesion vs. margin, margins vs. surrounding liver and center of the lesion vs. surrounding liver were found regarding WiAuC, mTT, Ri and TTP.

**Table 1:** Mean ±SD of pE, WiAuC, mTT, TTP and Ri each in the center of the lesion, the margins and the periphery, collected within 24 h following TACE.

	center (mean ± SD)	margin (mean ± SD)	Surrounding liver (mean ± SD)
**pE**	867.8 ± 2416	2028 ± 3954	2824 ± 4290
**WiAuC**	385.4 ± 1262	564.6 ± 954.9	1149 ± 4473
**mTT**	211.3 ± 195.0	184.1 ± 150.6	163.8 ± 157.1
**TTP**	24.15 ± 13.97	22.40 ± 11.70	20.93 ± 10.69
**Ri**	19.84 ± 13.51	18.08 ± 10.35	16.78 ± 9.91

**Fig 1:**
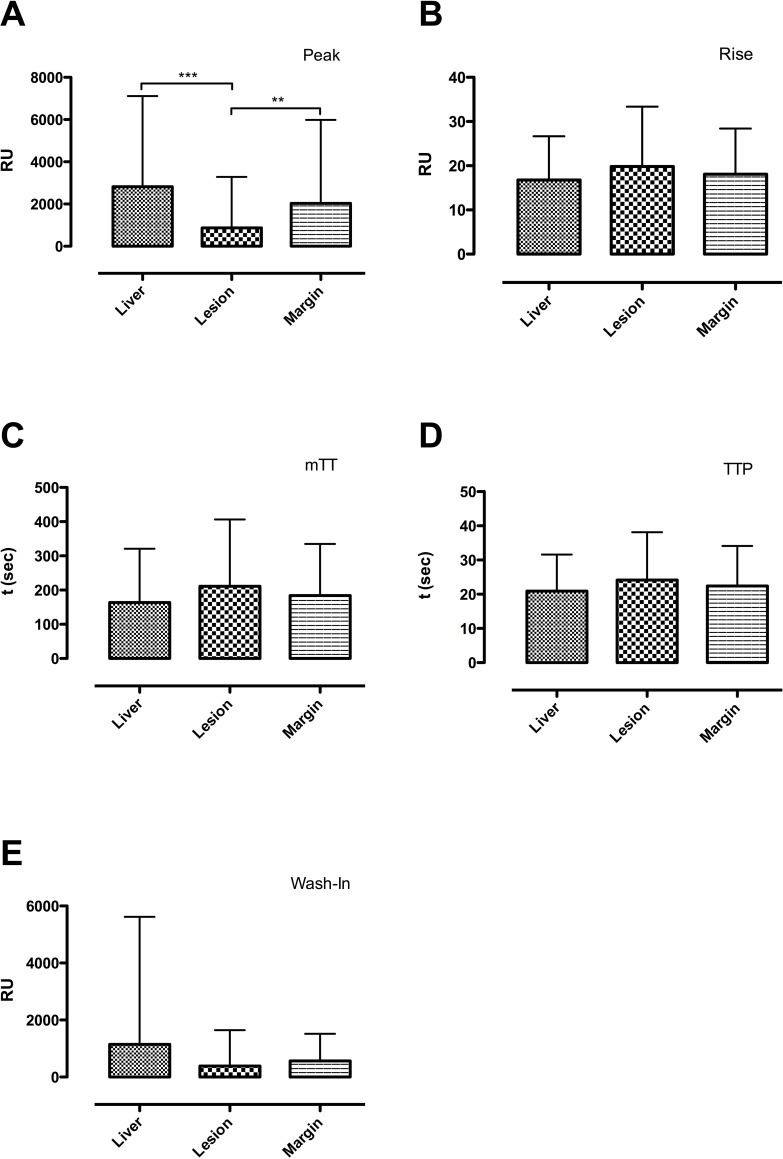
Comparison of all perfusion parameters. Significant differences were only observed for Peak (up left) when comparing the center of the lesion (center column) versus the surrounding liver tissue (left column) center of the lesion vs. its margins (right colum).

However, significant differences were found for pE center of lesion vs surrounding liver (p <0.0001) and center of lesion vs margin of lesion (p< 0.005). No significant differences were found for pE surrounding liver vs margin of lesion (Fig 1). The 95% confidence intervals (CI) of difference were 1067 to 2845 for center vs. surrounding liver, -92.86 to 1685 for surrounding liver vs. margin and -2049 to -271.4 for center vs. margin.

**Fig 2:**
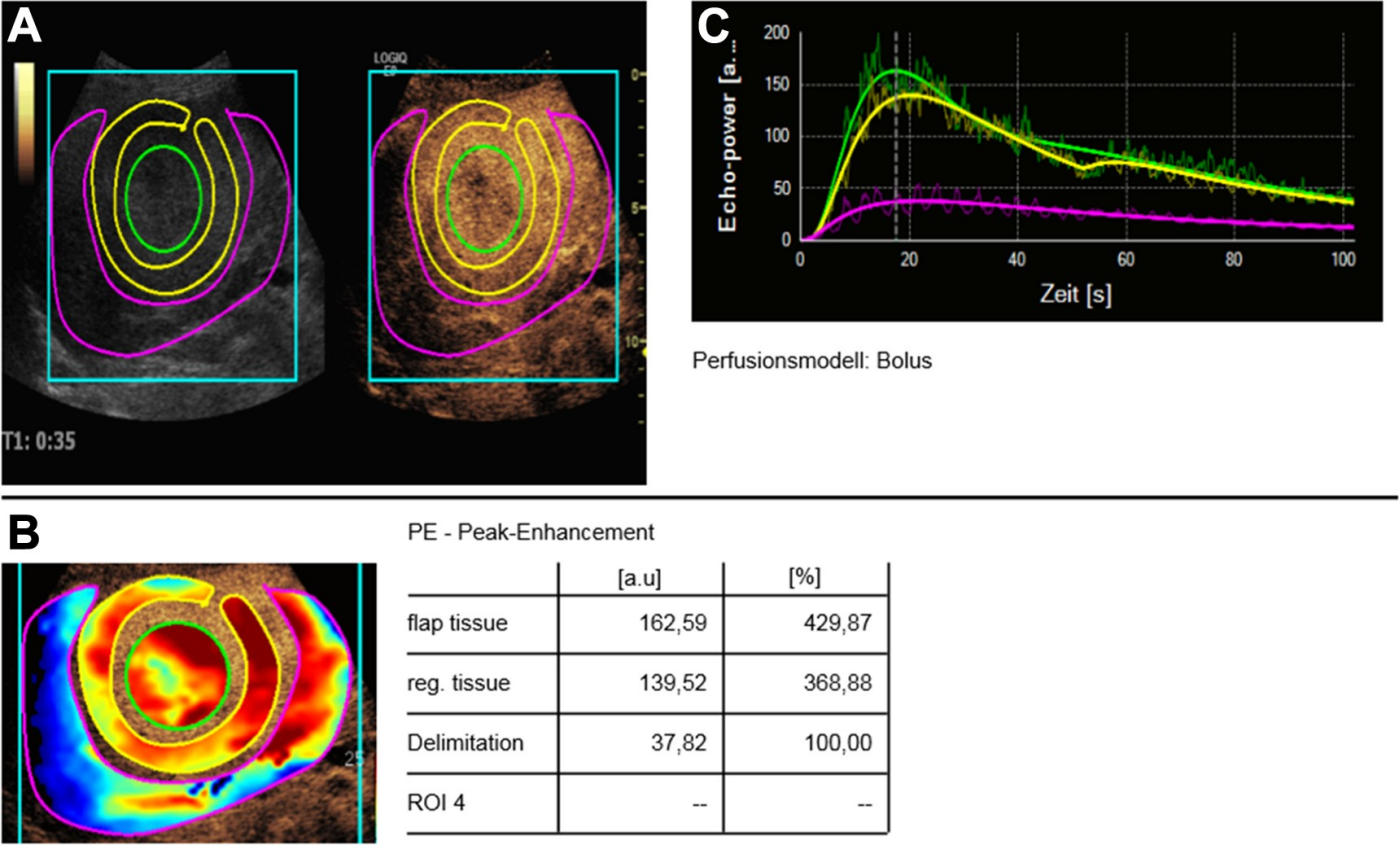
Pre-interventional evaluation for peak enhancement. A. Before embolization, the tumor and margin area show a profound enhancement in CEUS original images. B. In pseudo-colors the tumor is displayed in red colors. C. TIC analyses show that the perfusion curve for the center of the lesion (green) has a profound rise and fall consistent with a HCC.

**Fig 3:**
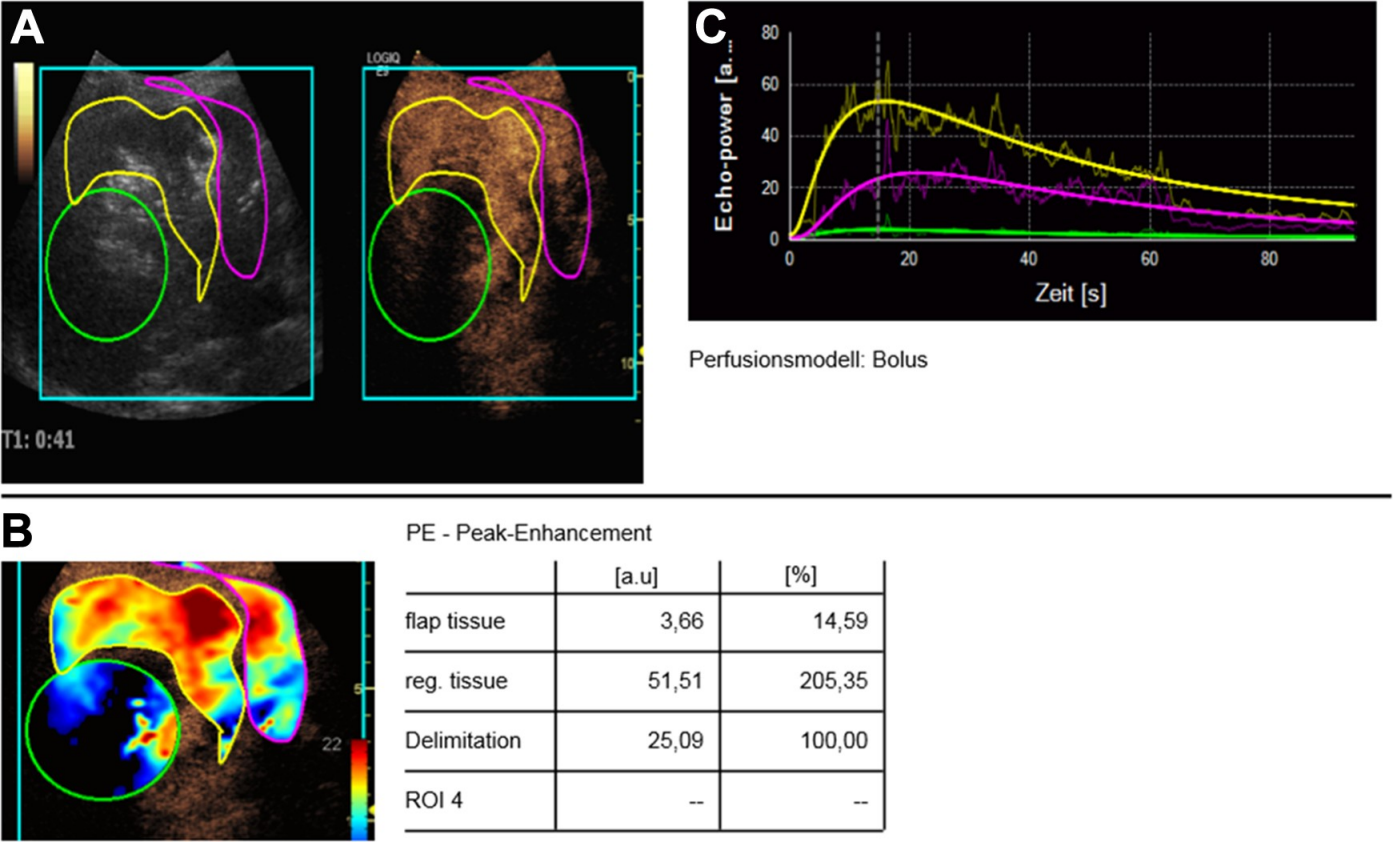
Case of successful TACE (display of peak enhancement). A. After the intervention, the post-embolization defect in CEUS original images appears black, meaning nearly avascular. B. In pseudo-colors the defect is shown in blue colors, also showing a devascularization. C. TIC analyses show that the perfusion curve for the center of the defect (green) is close to the baseline.

**Fig 4:**
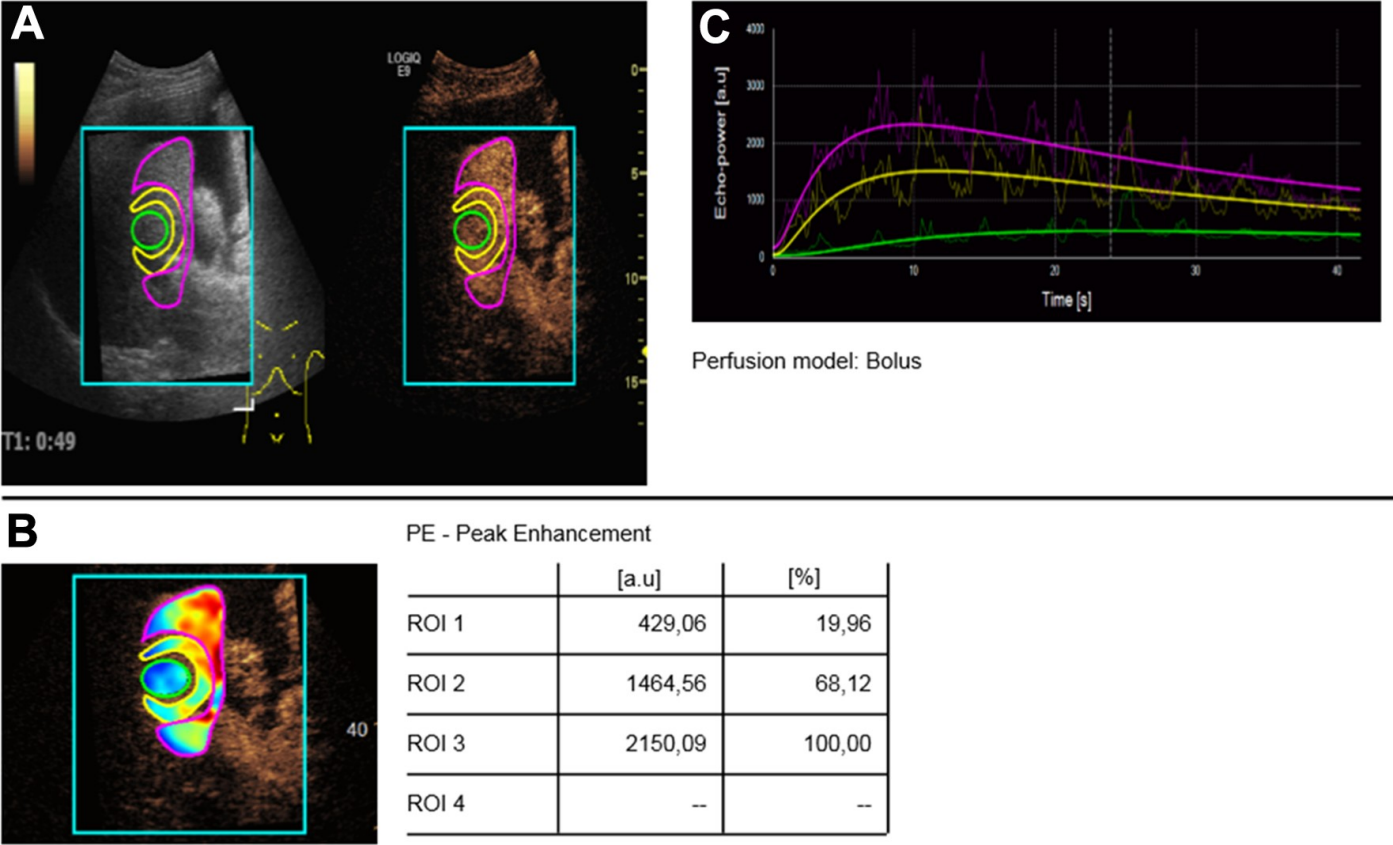
Case of a partially successful TACE (display of peak enhancement). A. The post-embolization defect in CEUS original images shows residual contrast enhancement, consistent with remaining tumor. B. In pseudo-colors the defect is shown in blue to green colors, proving an only partial devascularization. C. TIC analyses show that the perfusion curve for the center of the defect (green) is not quite close to the baseline confirming residual tumor.

The perfusion software uses pseudo-colors to show vascularization. Hypervascularization is shown in red and yellow shades (Fig 2). Devascularization is shown in blue and green colors. In all cases there was a profound visual reduction of vascularization displayed as blueish and greenish nuances (Figs 3 and 4).

## Discussion

Whereas surgical resection and ablation are widely accepted for curative care, trans-arterial chemoembolization remains the standard treatment option in patients with non-resectable HCC or if an ablative technique is not possible. The long-term results regarding tumor recurrence and overall survival are favorable compared to best supportive care [22]. Assessment of tumor response following interventional treatment is crucial for determination of treatment efficiency and further strategies.

Postinterventional success control following TACE and ablative techniques can be better evaluated by observing changes regarding the tumor vascularization rather than the size. Thus, CEUS is a valuable method for follow-up as it allows a continuous display of the microvascularization of a lesion [20, 23]. Previous CEUS studies have shown a sensitivity of 87–100% and a specificity of 65–100% in detecting residual tumor after TACE [24, 25]. Moschouris et al. used additionally perfusion quantification for HCC evaluation in both the pre- and post-TACE setting monitoring a statistically significant decrease in perfusion index (PI) after TACE [26].

The color coded perfusion software (VueBox) used in this study shows hyper enhancement in yellow and red shades, whereas devascularized areas appear blue. By using ROIs in the center of the lesion, the margins and the surrounding liver tissue the extent of devascularization could be analyzed. The therapeutic success postulates a near complete devascularization of the center of the malignant lesion as well as its border area. Nodular enhancement and irregular regional enhancement pattern correlate with residual vital tumor. However, contrast enhancement 24 hours after the ablation can either be evoked by reactive changes in terms of immunoresponses [27, 28] or remaining tumor. Thus, for TACE, the use of CEUS for follow-up within 24 hours post-intervention would not change the diagnostic and therapeutic setting. Previous studies have evaluated the perfusion parameters of gastrointestinal stoma tumors (GIST) [29] and prostate cancer [30]. However, this is the first time that a perfusion software was used for evaluation of the therapeutic success following TACE in HCC.

A critical point is, that the perfusion software often shows incomplete devascularization following TACE. Therefore, for establishing the clinical success, long- term follow-up is crucial. In this study, we were able to analyze and compare various perfusion parameters including peak enhancement, Wash-in area under the curve, mean transit time and time to peak in the center of the lesion, its border area and the periphery for the first time.

Our study had some limitations. The study population was heterogeneous with regard to the tumor sizes and the extent of liver cirrhosis. A further limitation is the still relatively small number of patients and lesions in the present study. Therefore, further studies with an increased number of patients are necessary. Also, the quality of CEUS examinations and thus VueBox evaluation strongly depends on the localization of the tumor lesion, as lesions in deeper layers are harder to visualize. Compared to other imaging modalities, CEUS requires specific technology and an experienced examiner and highly depend on the patient’s compliance regarding breathing and circulation.

However, due to the relatively low costs, compared to imaging modalities such as CT or MRI ultrasound and CEUS in particular is a valuable tool for repeating post-interventional follow-up examinations. Also, the contrast agents used in CEUS have a reduced risk profile in comparison to CT and MRI regarding renal and thyroid function and potential allergic risks.

In summary, it can be said that the contrast-enhanced ultrasound in combination with perfusion analysis is a valuable supporting tool for post-interventional success control following TACE of liver lesions.

## Supporting information

S1 Dataset Data for perfusion parameter evaluation following TACE. (XLSX)
